# Literary Notes

**Published:** 1909-07-10

**Authors:** 


### LITERARY NOTES.

The current issue of *Guy's Hospital Gazette* contains, in the form of a supplement, an excellent photogravure portrait of the late Dr. Peter Horrocks, obstetric physician to the hospital. This portrait has been reproduced from the last photograph taken of Dr. Horrocks, and is a very characteristic likeness. Copies will be sent for 6½d. post free to any address on application to the editor of the *Gazette.*

The report for 1908 of the Mission to Lepers in India and the East, received from the London Office, 33 Henrietta Street, W.C., gives an account of work in seventy-three stations and among 7,295 lepers. Over 600 untainted children of leprous parents are being educated in homes connected with the Society. The expenditure for the year was £28,882, of which £8,638 was received abroad, mainly in grants from the Indian Governments. This indicates that the Mission has gained the confidence and sympathy of the authorities. The Society's offer to admit the Chinese leper recently discovered in Cardiff into one of its Chinese asylums will probably be accepted.

